# Phytoplasma SAP11 alters 3-isobutyl-2-methoxypyrazine biosynthesis in *Nicotiana benthamiana* by suppressing *NbOMT1*

**DOI:** 10.1093/jxb/erw225

**Published:** 2016-06-08

**Authors:** Choon Meng Tan, Chia-Hua Li, Nai-Wen Tsao, Li-Wen Su, Yen-Ting Lu, Shu Heng Chang, Yi Yu Lin, Jyun-Cyuan Liou, Li-Ching Hsieh, Jih-Zu Yu, Chiou-Rong Sheue, Sheng-Yang Wang, Chin-Fa Lee, Jun-Yi Yang

**Affiliations:** ^1^Institute of Biochemistry, National Chung Hsing University, Taichung 402, Taiwan; ^2^Ph.D. Program in Microbial Genomics, National Chung Hsing University and Academia Sinica, Taiwan; ^3^Department of Forestry, National Chung Hsing University, Taichung 402, Taiwan; ^4^Department of Chemistry, National Chung Hsing University, Taichung 402, Taiwan; ^5^Institute of Genomics and Bioinformatics, National Chung Hsing University, Taichung 402, Taiwan; ^6^Department of Applied Zoology, Agricultural Research Institute, Taichung 413, Taiwan; ^7^Department of Life Sciences, National Chung Hsing University, Taichung 402, Taiwan; ^8^Institute of Biotechnology, National Chung Hsing University, Taichung 402, Taiwan; ^9^NCHU-UCD Plant and Food Biotechnology Center, National Chung Hsing University, Taichung 402, Taiwan

**Keywords:** 3-Isobutyl-2-methoxypyrazine, effector, *Nicotiana benthamiana*, *O*-methyltransferase, phytoplasma, SAP11, TCP transcription factor.

## Abstract

Phytoplasmas are bacterial phytopathogens that release virulence effectors into sieve cells and act systemically to affect the physiological and morphological state of host plants to promote successful pathogenesis. We show here that transgenic *Nicotiana benthamiana* lines expressing the secreted effector SAP11 from *Candidatus* Phytoplasma mali exhibit an altered aroma phenotype. This phenomenon is correlated with defects in the development of glandular trichomes and the biosynthesis of 3-isobutyl-2-methoxypyrazine (IBMP). IBMP is a volatile organic compound (VOC) synthesized by an *O*-methyltransferase, via a methylation step, from a non-volatile precursor, 3-isobutyl-2-hydroxypyrazine (IBHP). Based on comparative and functional genomics analyses, *NbOMT1*, which encodes an *O*-methyltransferase, was found to be highly suppressed in *SAP11*-transgenic plants. We further silenced *NbOMT1* through virus-induced gene silencing and demonstrated that this enzyme influenced the accumulation of IBMP in *N. benthamiana*. *In vitro* biochemical analyses also showed that NbOMT1 can catalyse IBHP *O*-methylation in the presence of S-adenosyl-L-methionine. Our study suggests that the phytoplasma effector SAP11 has the ability to modulate host VOC emissions. In addition, we also demonstrated that SAP11 destabilized TCP transcription factors and suppressed jasmonic acid responses in *N. benthamiana*. These findings provide valuable insights into understanding how phytoplasma effectors influence plant volatiles.

## Highlight

Phytoplasma effector SAP11 modulates plant volatile organic compound emissions by suppressing the expression of *NbOMT1*, which encodes an *O*-methyltransferase required for the biosynthesis of 3-isobutyl-2-methoxypyrazine.

## Introduction

Plants produce volatile organic compounds (VOCs) as chemical cues to communicate with associated community members. These VOCs can provide signals to locate hosts for attackers. For example, *Candidatus* Phytoplasma mali infection causes apple (*Malus* sp.) plants to release β-caryophyllene, which is considered to be an odour cue for attracting *Cacopsylla picta*, an insect vector of *Ca.* P. mali (Mayer *et al.*, 2008a, b). *Candidatus* Liberibacter asiaticus (Las) infection alters VOC emissions in citrus trees, thereby attracting *Tamarixia radiata* to increase the parasitism of *Diaphorina citri*, an insect vector of Las (Martini *et al.*, 2014). Therefore, VOCs emitted by pathogen-infected hosts are likely to influence the behaviour of subsequent attackers, including both insect vectors and the natural enemies of insect vectors. However, studies regarding the role of odour cues in the interactions between infected individuals and other organisms remain relatively few in number, and the molecular mechanisms remain unclear.

3-Alkyl-2-methoxypyrazines (MPs) are odorant VOCs with extremely low sensory detection thresholds that are found in many vegetables, fruit, and insects (Sidhu *et al.*, 2013). MPs have diverse biological functions and not only act as a warning signal to potential predators (Rothschild *et al.*, 1984; Moore *et al.*, 1990) but also play a role in pheromonal attraction between insects (Al Abassi *et al.*, 1998; Susset *et al.*, 2013; Wheeler and Cardé, 2013). In the wine industry, MPs have been associated with the vegetable-like flavour of certain wine varietals, in particular, Cabernet Sauvignon (Ebeler and Thorngate, 2009). Three MPs, 3-isobutyl-2-methoxypyrazine (IBMP), 3-isopropyl-2-methoxypyrazine, and 3-*sec*-butyl-2-methoxypyrazine, are present in grape berries (Ebeler and Thorngate, 2009; Sidhu *et al.*, 2013). Among them, IBMP has the highest concentration and is responsible for the distinct characteristics of wine.

It has been proposed that methylation of 3-alkyl-2-hydroxypyrazines, the precursors of MPs, is mediated by *O*-methyltransferases (OMTs) (Hashizume *et al.*, 2001). OMTs constitute a large family of enzymes that catalyse methylation of the hydroxyl groups of various secondary metabolites (Lam *et al.*, 2007; Barakat *et al.*, 2011). Phylogenetic analyses reveal that OMTs can be categorized into two main classes. One of the classes includes caffeoyl coenzyme A 3-*O*-methyltransferases (CCoAOMTs) and caffeic acid 3-*O*-methyltransferases, which methylate hydroxyl groups of phenylpropanoids. The other class includes the remaining OMTs, which methylate the hydroxyl groups of diverse metabolites, including flavonoids, alkaloids, and phytoalexins. Currently, only *VvOMT3* and *VvOMT4* have been identified in grapes (*Vitis vinifera*) as key genes that encode OMTs in the methylation of 3-isobutyl-2-hydroxypyrazine (IBHP), the precursor of IBMP (Dunlevy *et al.*, 2013; Guillaumie *et al.*, 2013).

Recent studies of pathogen effectors have revealed that vector-borne pathogens can alter plant chemicals and development in manners that affect the preference or performance of insect vectors. These effector-triggered changes in plant traits have significant implications for the transmission and spread of diseases (Yang *et al.*, 2008; Sugio *et al.*, 2011; Zhang *et al.*, 2012). In the past few years, multiple potentially secreted proteins have been predicted as candidate effectors in phytoplasmas through genome analysis. However, only a few of them have been characterized. Among them, SAP11, the secreted protein 11 of the Aster yellows witches’-broom phytoplasma (AY-WB) strain, has been reported to contain a bipartite nuclear localization signal required for nuclear targeting in plant cells (Bai *et al.*, 2008; Sugio *et al.*, 2014). This subcellular localization is responsible for the SAP11-mediated destabilization of class II CINCINNATA (CIN)-related TEOSINTE BRANCHED1, CYCLOIEDA, PROLIFERATING CELL FACTORs (TCPs) transcription factors, leading in turn to down-regulation of jasmonic acid (JA) biosynthesis and an increase in the progeny of the leafhopper vector *Macrosteles quadrilineatus* (Sugio *et al.*, 2011).

*Ca.* P. mali is an uncultivable bacterial pathogen that causes apple proliferation disease and is responsible for great economic losses in the apple industry (Seemüller and Schneider, 2004). It is believed that *Ca.* P. mali can change the VOCs of its host plant and its vector behaviours to promote the spread of apple proliferation disease (Mayer *et al.*, 2008a, 2008b). Given the importance of VOCs in the communication between plants and associated community members, we hypothesized that *Ca.* P. mali might release potential virulence factors into plant cells to manipulate VOC emissions. Genes that encode candidate secreted proteins have been predicted in the genome of *Ca.* P. mali, including a homologue of SAP11 (Kube *et al.*, 2014; Siewert *et al.*, 2014). In this study, we examined the effects of SAP11 on plant VOC emissions and developmental alterations.

## Materials and methods

### Plant materials and growth conditions

*N. benthamiana* was grown at 25°C in a semi-controlled walk-in chamber with a 16:8-h light to dark photoperiod for the generation of transgenic plants or agroinfiltration.

### Plasmid construction

All DNA manipulations were performed using standard restriction site reconstruction techniques and confirmed by DNA sequencing. For the generation of *SAP11*_*CaPM*_ -transgenic plants, a codon- optimized *SAP11*_*CaPM*_ sequence encoding a protein without the signal peptide was amplified using AccuPrime pfx DNA polymerase (Invitrogen) and subcloned into a pBA002 vector under the control of a *Cauliflower mosaic virus* (*CaMV*) 35S promoter. For the expression of FLAG-tagged TCPs (SFP-TCPs), an *N. benthamiana* cDNA library was used to amplify the *TCP*s and then subcloned into a pBA-N-SFP vector (Su *et al.*, 2013) under the control of the 35S promoter. To produce recombinant proteins, PCR products encoding SAP11_CaPM_ or NbOMT1 were subcloned into the pET-SUMO (Invitrogen) vector to produce N-terminal His-SUMO-tagged proteins. For the virus-induced gene silencing (VIGS) assay, a 185-bp DNA fragment located downstream of the *NbOMT1* coding sequence was amplified and subcloned into the pTRV2 vector (Ruiz *et al.*, 1998). The primers used are listed in Supplementary Table S1.

### Generation of transgenic plants

*Agrobacterium*-mediated transformations of *N. benthamiana* were performed by the leaf disc method using the *A. tumefaciens* strain ABI (Horsch *et al.*, 1985). To obtain transgenic lines, seedlings were selected on a half-strength Murashige and Skoog (1/2× MS) medium containing both Basta (25 μg mL^−1^) and carbenicillin (100 μg mL^−1^) and then examined via western blotting using specific anti-SAP11_CaPM_ antibodies.

### Antibody production and western blotting

His-SUMO-SAP11_CaPM_ recombinant protein was produced in *Escherichia coli* BL21 (DE3) cells at 24°C after isopropyl β-D-1-thiogalactopyranoside induction. The protein was purified using Ni^2+^-NTA resin (Qiagen) after cell lysis. To raise a specific antibody against SAP11_CaPM_, His-SUMO-SAP11_CaPM_ was cleaved with Ubl-specific protease 1 (Ulp1) to remove the His-SUMO tag, and the SAP11_CaPM_ protein was obtained using a Sephacryl S-200 HR gel filtration column (GE Healthcare). To detect SAP11_CaPM_, total cell extracts of *SAP11*_*CaPM*_-transgenic plants were prepared by directly adding 2.5× SDS sample buffer to ground samples. Western blotting was performed using enhanced chemiluminescence western-blotting reagents (Amersham), and chemiluminescence signals were captured using a ImageQuant LAS 4000 Mini (GE Healthcare).

### Quantitative reverse transcription PCR

To measure gene expression levels, total RNA was extracted from 6-week-old *N. benthamiana* using the TRIzol reagent. The cDNA was synthesized from 1 μg total RNA using Superscript III First-Strand Synthesis SuperMix (Invitrogen) according to the manufacturer’s instructions. The quantitative reverse transcription (qRT)-PCR reactions were performed on an Eco Real-Time PCR System (Illumina) using the KAPA SYBR FAST qPCR Kit (Kapa Biosystems) under the following conditions: 95°C for 1min followed by 40 cycles of 95°C for 15s, 58°C for 15s, and 72°C for 30s. The relative amounts of transcripts were determined by normalization to the reference gene, *Actin*. The primers are listed in Supplementary Table S1.

### Droplet digital PCR

The droplet digital (dd)PCR assays were conducted using the QX200 Droplet Digital PCR System (Bio-Rad) with the cDNA templates prepared for the qRT-PCR assays. The reaction mixture contained 10 μL of 2× Evagreen Digital PCR Supermix (Bio-Rad), 2 μL of 30× diluted cDNA template, and 500nM PCR primers in a final volume of 20 μL and was processed using a droplet generator (Bio-Rad) to generate thousands of nanolitre-sized droplets. PCRs were run under the following standard cycling conditions: 95°C for 10min, 40 cycles of 94°C for 30s and 58°C for 1min, 98°C for 10min, and a holding temperature of 4°C. After amplification, droplets were detected individually on a QX200 Droplet Reader, and the data were analysed using the QuantaSoft software package (Bio-Rad). The primers are listed in Supplementary Table S1.

### Co-expression assays

The co-expression assays were performed via agroinfiltration (Leuzinger *et al.*, 2013) with a mixture of *A. tumefaciens* strain ABI carrying plasmids constructed for the expression of SAP11_CaPM_ and FLAG-tagged TCPs in *N. benthamiana*. After 2 days, co-infiltrated leaves were harvested and ground for western blot analyses. The expression levels of SAP11_CaPM_ and FLAG-tagged TCPs were detected by specific antibodies against SAP11_CaPM_ and the FLAG tag.

### Headspace solid-phase microextraction/GC-MS analyses

To identify the VOCs released from plant leaves and *E. coli* culture medium, headspace solid-phase microextraction (HS-SPME) coupled to GC-MS was performed. Chipped leaves or culture supernatant was sealed in a headspace glass vial, and VOCs were collected via HS-SPME using a 75-μm carboxen-polydimethylsiloxane fibre (Sigma) at 60°C for 10min. After extraction, the SPME fibre was directly injected into a TRACE GC Ultra Gas Chromatograph (Thermo) coupled to an ITQ 900 mass spectrometer (Thermo) functioning in electron impact mode at 70eV. The analyses were performed on a DB-5ms column (30 m × 0.25mm inside diameter, 0.25-μm film thickness; J & W Scientific) using helium as a carrier gas with a flow rate of 1mL min^−1^. The injector temperature was 240°C. The oven temperature was set to 40°C for 5min, and the temperature was then increased to 170°C at a rate of 5°C min^−1^, followed by an increase to 280°C at a rate of 15°C min^−1^. The final temperature was held for 5min. Mass spectra were acquired within a mass-to-charge range of 40–600 *m/z*. Eluting compounds were identified via comparison to the Wiley Registry/NIST Mass Spectral Library. An authentic standard (IBMP) was purchased from Sigma to confirm the retention time and mass spectrum. The relative amounts of IBMP between samples were obtained after normalizing to an internal standard (bornyl acetate).

### In vitro O-methylation assays

IBHP was chemically synthesized. Recombinant proteins were immobilized on Ni^2+^-NTA resin by adding 10 μL pre-washed beads to 2mL of crude *E. coli* BL21 (DE3) cell extracts and incubating at 4°C for 1h. After washing (50mM Tris-HCl pH 7.4, 20mM imidazole), 1mL of reaction buffer (50mM Tris-HCl pH 7.4, 2mM DTT, 0.05mM IBHP, 0.8mM S-adenosyl-L-methionine [SAM]) was added to each sample, and enzymatic reactions were performed in a Thermomixer C (Eppendorf) at 26°C with a stirring speed of 800rpm. After 12h, the reactions were terminated by adding 10 μL of 2M HCl, and the mixtures were extracted with 0.2mL of ethyl acetate. The organic phase products were injected into an Agilent 6890N Network GC coupled with an Agilent 5 973 Network Mass Selective Detector functioning in electron impact mode at 70eV. The injected sample (1 μL) was separated on an HP-INNOWax capillary column (30 m × 0.25mm × 0.25 μm) using helium as a carrier gas with a flow rate of 1mL min^−1^. The injector temperature was 250°C. The oven temperature was set to 50°C and then increased to 280°C at a rate of 20°C min^−1^. Mass spectra were acquired within a mass-to-charge range of 0–350 *m/z*. The IBMP was confirmed by comparison with the authentic standard.

### Cryo-SEM

Fresh leaves from 4-week-old *N. benthamiana* were dissected and loaded into a stub. After freezing with liquid nitrogen slush, samples were transferred to a preparation chamber at −160°C. The temperature was then increased to −85**°**C, and the samples were etched for 15min. After etching, the samples were coated with platinum at −130°C and then transferred to the SEM chamber. Images were obtained at −160°C using a Cryo-SEM (FEI Quanta 200 SEM/Quorum Cryo System PP2000TR FEI) at 20kV.

### VIGS assays

Plasmids (pTRV1, pTRV2, and pTRV2-*NbOMT1*) were individually introduced into the *A. tumefaciens* strain ABI via the freeze–thaw method. *Agrobacterium* cultures with an OD_600_ = 1 were incubated in 150 μM acetosyringone and 10mM MgCl_2_ for 2h at room temperature. Before infiltration, pTRV2 or pTRV2-*NbOMT1* containing *A. tumefaciens* was mixed with pTRV1 containing *A. tumefaciens* at a 1:1 (v:v) ratio and infiltrated into the lower leaves of four-leaf-stage plants using a 1-mL needleless syringe. After 3 weeks, the upper leaves of the infiltrated plants were collected for qRT-PCR and HS-SPME/GC-MS analyses.

## Results

### Phytoplasma SAP11_CaPM_ expression causes morphological changes in *N. benthamiana*

**Fig. 1.**
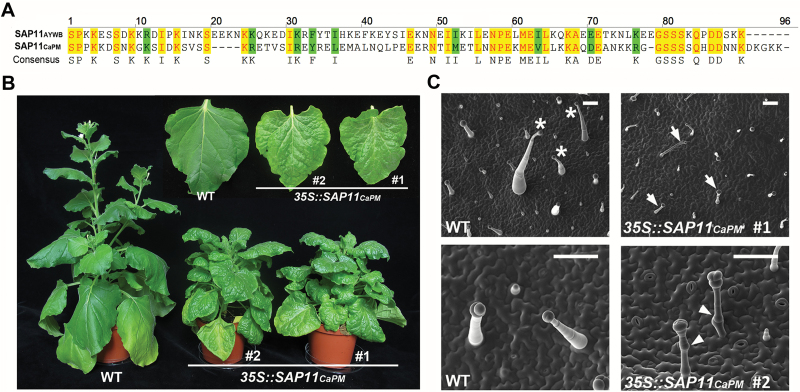
Investigation of morphological changes caused by SAP11_CaPM_ in *N. benthamiana*. (**A**) The alignment of amino acid sequences of SAP11_AYWB_ and SAP11_CaPM_ without the signal peptide. Identical residues are shaded in yellow, and similar residues are shaded in green. (**B**) Growth phenotypes of 10-week-old WT and *SAP11*_*CaPM*_-transgenic *N. benthamiana*. The upper part of the image shows an enlarged view of leaves. (**C**) Cryo-SEM images of trichomes on the adaxial leaf surface in WT and *SAP11*_*CaPM*_-transgenic *N. benthamiana*. Multicellular glandular trichomes with bamboo shoot-shaped and bamboo stalk-shaped structures are indicated by asterisks and arrows, respectively. The node-like structures between cell junctions of glandular trichomes are indicated by arrowheads. The scale bars correspond to 100 µm.

A protein sequence comparison revealed that SAP11_CaPM_ (a SAP11 homologue of *Ca.* P. mali) shares 36% identity and 56% similarity in amino acid sequences with SAP11_AYWB_ (AY-WB phytoplasma SAP11) (Fig. 1A). To investigate the function of SAP11_CaPM_, transgenic *N. benthamiana* lines expressing *SAP11*_*CaPM*_ under the control of a *CaMV* 35S promoter were generated. The *SAP11*_*CaPM*_-transgenic lines exhibited clear morphological changes with crinkled leaves and dwarf phenotypes (Fig. 1B). Interestingly, a glossy surface with bright green coloration was observed in *SAP11*_*CaPM*_-transgenic line leaves compared to wild type (WT) *N. benthamiana* (Fig. 1B). Further examination under a cryo-SEM revealed that the most obvious difference caused by SAP11_CaPM_ expression was in the multicellular glandular trichome phenotype (Fig. 1C), although an approximately 15% reduction of the total trichome number was observed in the *SAP11*_*CaPM*_-transgenic lines (see Supplementary Fig. S1 at *JXB* online). Compared to WT *N. benthamiana*, tall glandular trichomes with bamboo shoot-shaped structures were completely absent in the *SAP11*_*CaPM*_-transgenic lines, and only short glandular trichomes with bamboo stalk-shaped structures were observed (Fig. 1C). Moreover, abnormal morphology, consisting of node-like structures between cell junctions on the short glandular trichome stalks, only appeared in *SAP11*_*CaPM*_-transgenic lines (Fig. 1C). Because glandular trichomes can produce and retain volatile compounds on leaf surfaces, the morphological alterations in glandular trichomes suggest that SAP11_CaPM_ might affect plant VOC emissions.

### Phytoplasma SAP11_CaPM_ expression dramatically suppresses IBMP accumulation in *N. benthamiana*

**Fig. 2.**
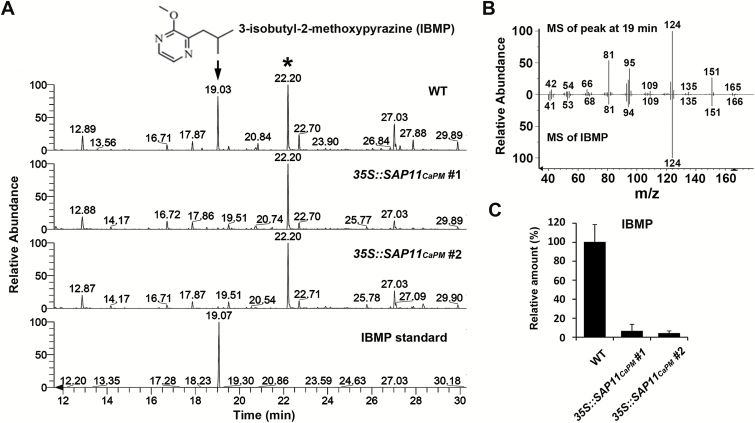
SAP11_CaPM_ suppresses IBMP accumulation in *N. benthamiana*. The emission profiles of VOCs released from the leaves of WT and *SAP11*_*CaPM*_-transgenic *N. benthamiana* were determined using HS-SPME coupled GC-MS. The significantly decreased compound (arrow) in *SAP11*_*CaPM*_-transgenic *N. benthamiana* was identified through comparison of its retention time (**A**) and mass fragmentation profile (**B**) with an authentic standard, IBMP. The internal standard, bornyl acetate, is indicated by an asterisk. The relative amount of IBMP shown in the chromatograms in A was calculated and is presented in **C**.

To examine whether SAP11_CaPM_ can affect the emission of VOCs in *N. benthamiana*, VOCs released from 8-week-old plant leaves were collected via HS-SPME and analysed using GC-MS. The analysis of the resulting chromatograms revealed that, at a retention time of 19min, a clear peak was visible in the VOC emission profile of WT *N. benthamiana* but hardly detected in transgenic lines expressing SAP11_CaPM_ (Fig. 2A). Based on a comparison with a published database (Wiley Registry/NIST Mass Spectral Library), the mass spectrum of the differentially expressed VOC (peak at 19min) identified in the gas chromatograms matched the IBMP mass spectrum (Fig. 2B). Further investigation with the authentic standard confirmed that this particular VOC, with a retention time of 19min in gas chromatograms, was IBMP (Fig. 2A). After normalization with an internal standard (bornyl acetate), the relative proportion of IBMP in WT and transgenic *N. benthamiana* was approximately 20:1 (Fig. 2C). These results indicate that IBMP accumulation is strongly suppressed by phytoplasma SAP11_CaPM_ expression in *N. benthamiana*.

### Phytoplasma SAP11_CaPM_ expression blocks *NbOMT1* expression in *N. benthamiana*

**Fig. 3.**
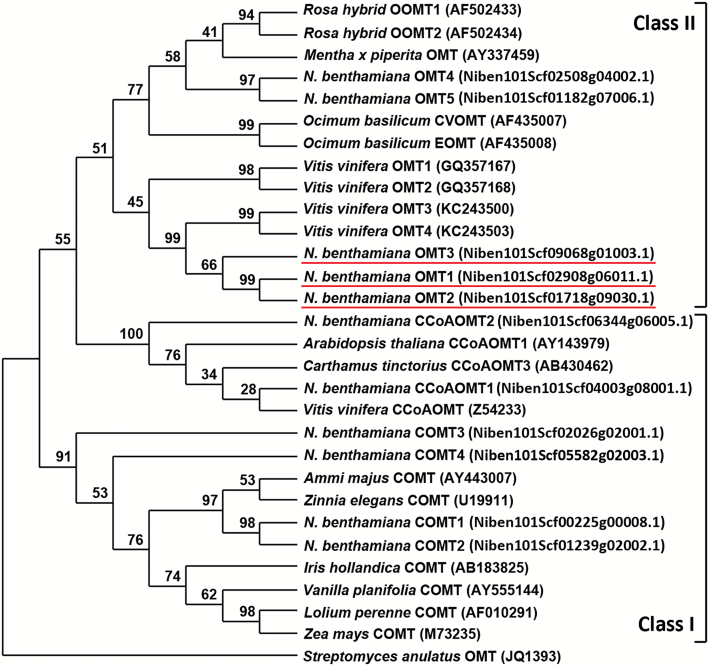
Phylogenetic analysis of *N. benthamiana* OMTs with other previously characterized plant OMTs (class II), caffeic acid 3-*O*-methyltransferases (COMTs; class I), and caffeoyl coenzyme A 3-*O*-methyltransferases (CCoAOMTs; class I). An outgroup OMT from *Streptomyces anulatus* was used. The numbers at the branch points are bootstrap values that represent the percentage of replicate trees based on 1000 repeats. NbOMT1, NbOMT2, and NbOMT3 are underlined. This figure is available in colour at *JXB* online.

**Fig. 4.**
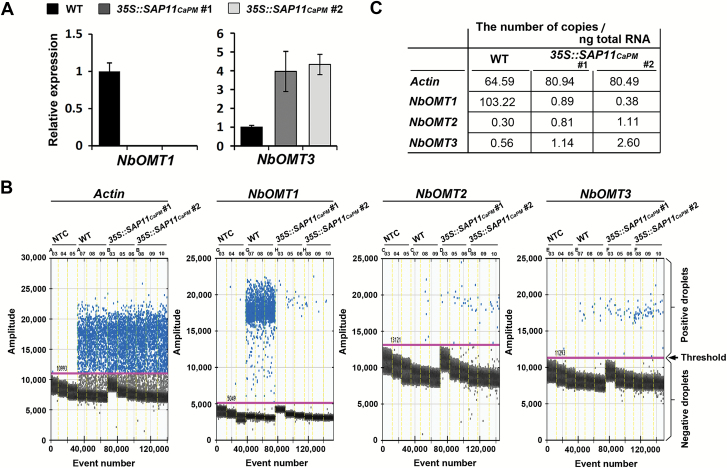
SAP11_CaPM_ represses *NbOMT1* expression in *N. benthamiana*. (**A**) Examination of the transcript levels of class II *OMT* genes by qRT-PCR. The relative gene expression levels in WT *N. benthamiana* were set to 1 after normalizing to *Actin*. (**B**) Precise measurements of gene transcript levels by ddPCR. The output levels for the detection of gene transcripts in WT and *SAP11*_*CaPM*_-transgenic *N. benthamiana* are presented based on three replicates. Each dot represents a unique droplet, the event number represents the number of droplets counted in the wells over time, and the amplitude represents the fluorescence signal detected in each droplet. The thresholds (pink lines) were set manually based on results from the non-template control (NTC). Droplets over the threshold are classified as positive (blue), and droplets blow the threshold are classified as negative (black). (**C**) Summary of the ddPCR data quantification. The original data (number of copies per μL of PCR mixture) for the concentration of target molecules were recalculated and are presented as the number of copies per ng total RNA.

OMTs have been demonstrated to be key players involved in IBMP biosynthesis in grapes (Dunlevy *et al.*, 2013; Guillaumie *et al.*, 2013). To identify the OMTs responsible for IBMP loss in *SAP11*_*CaPM*_-transgenic lines, a BLAST search against the *N. benthamiana* genome from the SOL Genomics Network (http://sgn.cornell.edu) was performed for VvOMT3, an essential OMT required for the final step of IBMP biosynthesis in grapevines. Through a comparative analysis, three *VvOMT3*-like genes termed *NbOMT1*, *NbOMT2*, and *NbOMT3* were identified in *N. benthamiana* (see Supplementary Fig. S2 at *JXB* online). Phylogenetic comparisons of NbOMT1, NbOMT2, and NbOMT3 to published plant OMTs revealed that they belong to class II OMTs (Fig. 3). qRT-PCR analysis further revealed that only *NbOMT1* expression was down-regulated to a barely detectable level in *SAP11*_*CaPM*_-transgenic lines compared to WT *N. benthamiana*, whereas the transcript levels of *NbOMT3* were not decreased in *SAP11*_*CaPM*_-transgenic lines (Fig. 4A). However, it was difficult to detect the expression of *NbOMT2* by qRT-PCR, even in WT *N. benthamiana*. To clearly distinguish the expression levels between *NbOMT1*, *NbOMT2*, and *NbOMT3* in *N. benthamiana*, an absolute quantification of gene expression was conducted by ddPCR (Fig. 4B). With the molecular counting platform, we found that the transcript level of *NbOMT1* was 200- to 300-fold greater than for *NbOMT2* and *NbOMT3* in WT *N. benthamiana* (Fig. 4C). However, *NbOMT1* expression was reduced to the same level as *NbOMT2* and *NbOMT3* (or even lower) in the *SAP11*_*CaPM*_-transgenic lines (Fig. 4C). In contrast, the transcript levels of *NbOMT2* and *NbOMT3* increased by 3- to 4-fold in the *SAP11*_*CaPM*_-transgenic lines compared to WT *N. benthamiana* (Fig. 4C). The increases in the transcript levels of *NbOMT2* and *NbOMT3* were likely due to the functional redundancy among *NbOMT*s in *N. benthamiana*, even though the biosynthesis of IBMP remained impaired in the *SAP11*_*CaPM*_-transgenic lines.

### Down-regulation of NbOMT1 represses IBMP biosynthesis in N. benthamiana

**Fig. 5.**
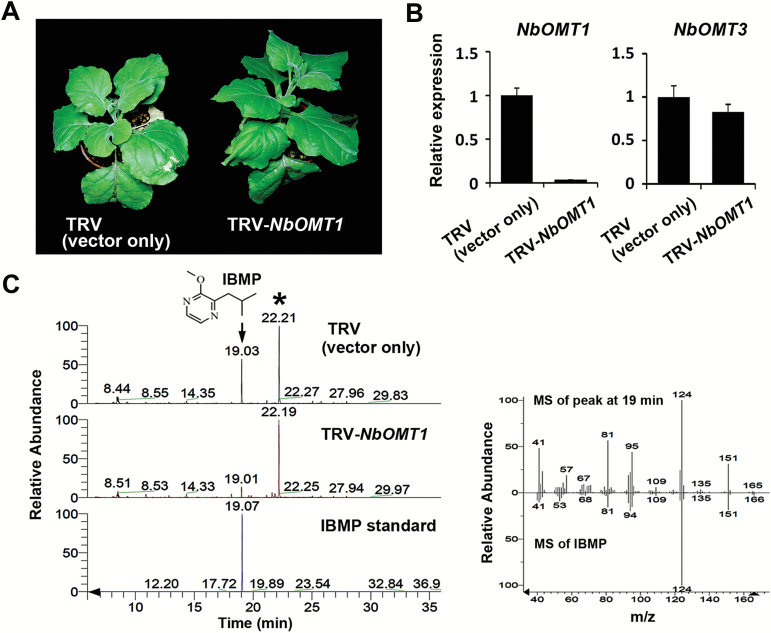
*NbOMT1* silencing represses IBMP production in *N. benthamiana*. (**A**) Comparison of the growth phenotypes between TRV- and TRV*-NbOMT1*-infected *N. benthamiana*. (**B**) Examination of the transcript levels of *OMT* genes by qRT-PCR. The relative gene expression levels in TRV-infected *N. benthamiana* were set to 1 after normalizing to *Actin*. (**C**) HS-SPME coupled GC-MS analyses of VOCs released from the TRV- and TRV*-NbOMT1* infected *N. benthamiana*. The significantly decreased compound (arrow) in *NbOMT1*-silenced *N. benthamiana* was identified by comparison of retention time with an authentic standard, IBMP. The internal standard, bornyl acetate, is indicated by an asterisk. Mass fragmentation profiles of the peak indicated by an arrow and IBMP are shown in the right panel.

To investigate the specific contribution of *NbOMT1* to IBMP production in *N. benthamiana*, a tobacco rattle virus (TRV)-based VIGS approach was used to transiently suppress *NbOMT1* expression. Here, a 185-bp fragment of *NbOMT1* was cloned into pTRV2 (see Supplementary Fig. S2 at *JXB* online), and a mixture of *Agrobacterium* cultures containing pTRV1 and pTRV2-*NbOMT1* (TRV*-NbOMT1*) was infiltrated into the lower leaves of four-leaf WT *N. benthamiana* plants. After 3 weeks, no phenotypic difference was observed between TRV (TRV1+TRV2, vector only)-infected and TRV*-NbOMT1*-infected *N. benthamiana* (Fig. 5A). Based on a qRT-PCR analysis, only *NbOMT1* expression was significantly reduced in the TRV*-NbOMT1*-treated plants compared to the non-silenced plants treated with a TRV vector only (Fig. 5B). To examine whether *NbOMT1* reduction affects IBMP production in *N. benthamiana*, VOCs released from the upper leaves of TRV or TRV-*NbOMT1* infiltrated plants were collected and analysed via HS-SPME coupled to GC-MS. Based on the resulting chromatograms, the peak that appeared at a retention time of 19min, with a mass spectrum that matched IBMP, was significantly reduced in TRV-*NbOMT1*-infiltrated plants (Fig. 5C). These results suggest that *NbOMT1* is primarily responsible for the biosynthesis of IBMP in *N. benthamiana*.

### NbOMT1 catalyses IBMP formation

**Fig. 6.**
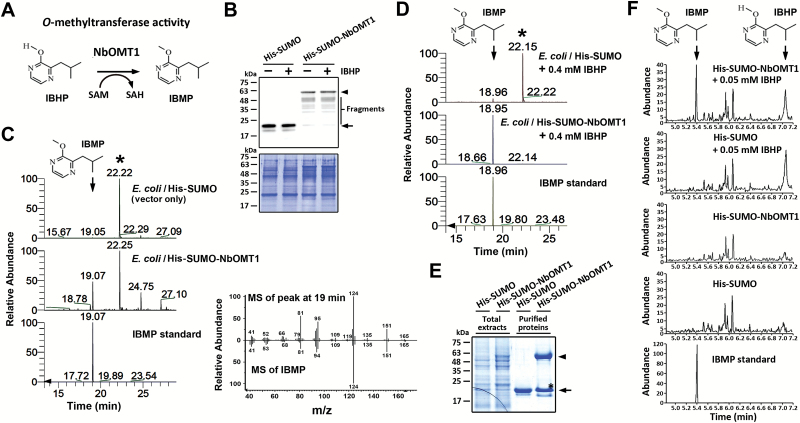
NbOMT1 possesses the ability to catalyse IBHP *O*-methylation. (**A**) A putative scheme for IBHP *O*-methylation by NbOMT1. (**B**) The expression levels of recombinant proteins were examined by western blotting using the specific antibody against the His tag. Coomassie Blue staining was provided as a loading control. The arrowhead indicates His-SUMO-NbOMT1; the arrow indicates His-SUMO. (**C**) HS-SPME coupled GC-MS analyses of VOCs released from the *E. coli* culture supernatants without adding synthetic IBHP. The significantly increased compound (arrow) in the His-SUMO-NbOMT1-expressing culture supernatant was identified through comparison of the retention time and mass fragmentation profile with an authentic standard, IBMP. The internal standard, bornyl acetate, is indicated by an asterisk. (**D**) HS-SPME coupled GC-MS analyses of VOCs released from the *E. coli* culture supernatants supplied with 0.4mM IBHP. The internal standard, bornyl acetate, is indicated by an asterisk. (**E**) Coomassie Blue staining of the SDS-PAGE analysis of recombinant proteins purified by Ni^2+^-NTA resin from *E. coli* cell extracts. The arrowhead indicates His-SUMO-NbOMT1, the arrow indicates His-SUMO, and the degradation product of His-SUMO-NbOMT1 is indicated by an asterisk. (**F**) GC-MS chromatograms of products extracted from the reaction mixtures of *in vitro* OMT activity assays. Enzyme reactions were conducted using recombinant proteins in the presence of SAM with or without IBHP. This figure is available in colour at *JXB* online.

To investigate the OMT activity accountable for IBMP production (Fig. 6A), *NbOMT1* was cloned into a pET vector and expressed in *E. coli* as a His-SUMO fusion recombinant protein (Fig. 6B). Unexpectedly, a vegetable-like odour was noted in the culture media of *E. coli* expressing His-SUMO-NbOMT1 without the addition of synthetic IBHP. The examination of culture supernatants using HS-SPME coupled to GC-MS revealed that His-SUMO-NbOMT1 expression resulted in the release of IBMP from the *E. coli* culture media (Fig. 6C). Further examination of IBMP production in culture media supplied with synthetic IBHP revealed 6600-times more IBMP in the culture media of *E. coli* expressing His-SUMO-NbOMT1 than in that of the His-SUMO control (Fig. 6D). To examine the OMT activity of NbOMT1 *in vitro*, recombinant proteins were purified from *E. coli* cell extracts (Fig. 6E) and incubated with IBHP in the presence of SAM. After incubation, the reaction mixtures were extracted using ethyl acetate, and the organic phase products were analysed using GC-MS without HS-SPME collection. Compared with the His-SUMO control, His-SUMO-NbOMT1 was able to methylate IBHP, thereby leading to production of IBMP (Fig. 6F). In contrast, no IBMP was detected when recombinant proteins were incubated without IBHP. These results indicate that NbOMT1 possesses the ability to catalyse IBHP *O*-methylation in the presence of SAM.

### Phytoplasma SAP11_CaPM_ destabilizes TCP transcription factors and suppresses JA responses in *N. benthamiana*

**Fig. 7.**
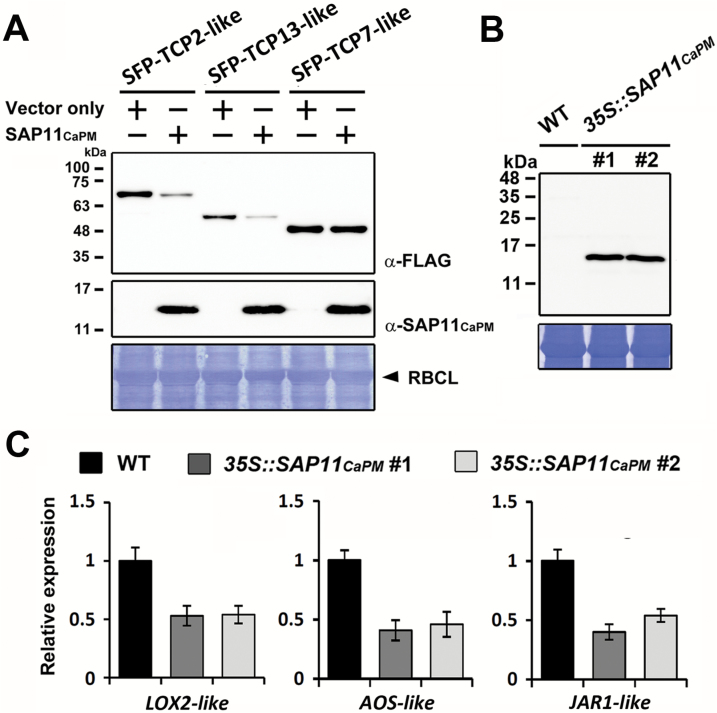
SAP11_CaPM_ destabilizes class II TCPs and alters the transcript levels of the genes involved in the JA response pathway in *N. benthamiana*. (**A**) Investigation of the relative abundance of *N. benthamiana* TCPs in the presence of SAP11_CaPM_ through transient co-expression assays. The expression levels of FLAG-tagged TCP2-like (Niben101Scf03932g05003.1), TCP13-like (Niben101Scf00757g05005.1), and TCP7-like (Niben101Scf09969g00001.1) proteins were examined by western blotting using the antibody against the FLAG tag. (**B**) Examination of SAP11_CaPM_ expression levels in transgenic *N. benthamiana* by western blotting using the antibody against SAP11_CaPM_. Rubisco large subunit (RBCL) stained with Coomassie Brilliant Blue was used as a loading control. (**C**) Examination of gene transcript levels in the JA response pathway by qRT-PCR. The relative expression levels of *LOX2-like* (Niben101Scf02688g02014.1), *AOS-like* (Niben101Scf05799g02010.1), and *JAR1-like* (Niben101Scf01076g00005.1) in WT *N. benthamiana* were set to 1 after normalizing to *Actin*. This figure is available in colour at *JXB* online.

It has been demonstrated that SAP11_AYWB_ can interact with and destabilize class II TCP transcription factors, thus leading to down-regulation of JA biosynthesis (Sugio *et al.*, 2011; Sugio *et al.*, 2014). To examine whether SAP11_CaPM_ can destabilize *N. benthamiana* TCPs, FLAG-tagged TCPs (SFP-TCPs) were transiently co-expressed with SAP11_CaPM_ in *N. benthamiana* leaves by agroinfiltration. Compared with the vector alone, the protein levels of class II TCPs (TCP2-like, TCP13-like) were greatly decreased in the presence of SAP11_CaPM_, whereas the abundance of class I TCPs (TCP7-like) was not affected (Fig. 7A). We further examined the transcript levels of genes involved in the JA-response pathway by qRT-PCR in *SAP11*_*CaPM*_-transgenic lines. With the expression of SAP11_CaPM_ (Fig. 7B), the transcript levels of *N. benthamiana LOX2-like*, *AOS-like*, and *JAR1-like* genes were decreased in the *SAP11*_*CaPM*_-transgenic lines compared with the WT control (Fig. 7C). These results suggest that SAP11_CaPM_ exhibits similar functions to SAP11_AYWB_ in destabilizing a TCP subset.

## Discussion

TCPs are well-known transcription factors that are involved in diverse developmental processes, including leaf development, branching, organ identity, senescence, cell proliferation, and germination (Martín-Trillo and Cubas, 2010; Manassero *et al.*, 2013). In this study, we demonstrated that SAP11_CaPM_ can destabilize a subset of TCPs in *N. benthamiana* (Fig. 7A). Based on the evidence that *tcp* mutants are also deficient in trichome development (Ori *et al.*, 2007; Hao *et al.*, 2012; Li *et al.*, 2012; Aguilar-Martínez and Sinha, 2013; Wang *et al.*, 2013) and that glandular trichomes are known to be able to produce, store, and secret secondary metabolites (Glas *et al.*, 2012; Tissier, 2012), it is possible that the destabilization of a subset of TCPs by SAP11_CaPM_ might cause glandular trichome structural alterations that in turn affect VOC emissions in *N. benthamiana*. However, through comparative analysis, 69 unigenes encoding putative TCP transcription factors were found in the *N. benthamiana* genome (see Supplementary Fig. S3 at *JXB* online). Thus, it will be challenging to characterize whether TCPs can regulate secondary metabolite biosynthesis and glandular trichome development in *N. benthamiana*.

Although the molecular mechanism underlying the SAP11_CaPM_-mediated loss of volatiles in *N. benthamiana* remains unclear, we demonstrated that SAP11_CaPM_ can reduce JA biosynthesis and responses through the down-regulation of *LOX2-like*, *AOS-like*, and *JAR1-like* genes (Fig. 7C). Among these genes, the *LOX2-like* gene encodes an enzyme that catalyses the formation of 13-hydroperoxylinolenic acid (13-HPOT), a substrate required for JA biosynthesis and the formation of green leaf volatiles (GLVs) such as (*Z*)-3-hexenal, (*Z*)-3-hexen-1-ol, and (*Z*)-3-hexenyl acetate (Halitschke *et al.*, 2004; Christensen *et al.*, 2013; Savchenko *et al.*, 2013). Thus, it is possible that the biosynthesis of GLVs is also impaired in *SAP11*_*CaPM*_-transgenic *N. benthamiana* lines. However, 13-HPOT is not a substrate required for IBMP biosynthesis (Dunlevy *et al.*, 2013; Guillaumie *et al.*, 2013). As a result, the dramatic decrease in IBMP emission should not be an indirect consequence of the suppression of JA synthesis in *SAP11*_*CaPM*_-transgenic *N. benthamiana*. Whereas the impact of JA and GLVs in affecting the fitness of phytoplasma insect vectors was not investigated here, it has been demonstrated that *N. attenuata* plants with decreased JA biosynthesis are more attractive to *Empoasca* leafhoppers, which are also phytoplasma insect vectors (Kallenbach *et al.*, 2012).

In addition to the dramatic reduction in tobacco volatiles, *SAP11*_*CaPM*_-transgenic *N. benthamiana* also exhibited a reduction in glandular trichomes and structural alterations (Fig. 1C; Supplementary Fig. S1). These changes are always accompanied by the phenomenon of bright green leaves with a glossy surface in *SAP11*_*CaPM*_-transgenic *N. benthamiana* (Fig. 1B). Although it remains unclear whether this structural colour plays a role in attracting insect herbivores (Glover and Whitney, 2010), it is believed that the defects in trichome production reduce the physical and chemical barriers required for insect herbivore resistance (Glas *et al.*, 2012; War *et al.*, 2012; Hauser, 2014). For example, the non-glandular trichome density on natural *Arabidopsis* populations is correlated with the oviposition resistance of *Plutella xylostella* (Handley *et al.*, 2005; Sletvold *et al.*, 2010). Moreover, the tomato (*Solanum lycopersicum*) *odorless-2* and *hairless* mutants exhibit defects in glandular trichome development and anti-insect metabolite accumulation, resulting in increased susceptibility to *Manduca sexta* (Kang *et al.*, 2010a, 2010b). This raises the possibility that the failure to produce certain glandular trichomes in *SAP11*_*CaPM*_-transgenic *N. benthamiana* may influence the fitness of the phytoplasma insect vector.

Phytoplasmas are mycoplasma-like bacterial pathogens associated with numerous plant diseases and are responsible for serious losses in agricultural productivity worldwide (Lee *et al.*, 2000). In the past decade, the whole-genome sequences for several phytoplasma strains have been reported (Bai *et al.*, 2006; Kube *et al.*, 2008; Tran-Nguyen *et al.*, 2008; Chung *et al.*, 2013; Chen *et al.*, 2014), beginning with the publication of the complete genomic sequence of the onion yellows phytoplasma strain M (OY-M) in 2004 (Oshima *et al.*, 2004). Through genomic characterization, up to 56 candidate effectors with an N-terminal signal peptide were predicted in a phytoplasma (Bai *et al.*, 2008). These secreted proteins are potential virulence factors, and some of them have been demonstrated to modulate plant development (Hoshi *et al.*, 2009; MacLean *et al.*, 2011; Sugio *et al.*, 2011; Maejima *et al.*, 2014), suppress plant defence response (Lu *et al.*, 2014), alter host nutrient environments (Lu *et al.*, 2014), and promote insect vector colonization (Sugio *et al.*, 2011; MacLean *et al.*, 2014). Here, we found that expression of the secreted effector SAP11_CaPM_ can inhibit IBMP biosynthesis via suppressing *NbOMT1* expression in *N. benthamiana* (Figs 2A, 4A). Considering the importance of IBMP, which not only acts as a warning signal to potential predators (Rothschild *et al.*, 1984; Moore *et al.*, 1990) but also plays a role in pheromonal attraction between insects (Al Abassi *et al.*, 1998; Susset *et al.*, 2013; Wheeler and Cardé, 2013), it is reasonable to assume that the SAP11_CaPM_-induced changes in host volatiles may have an impact on the attraction of phytoplasma insect vectors. However, this remains to be experimentally proven. Nevertheless, our study contributes to an improved understanding of the role of the phytoplasma effector SAP11_CaPM_ in influencing plant volatiles and may provide new insights into understanding the interactions between phytoplasmas and host plants.

## Supplementary data

Supplementary data are available at *JXB* online.

Fig. S1. Cryo-SEM images of trichomes on the adaxial leaf surface in WT and *SAP11*_*CaPM*_-transgenic *N. benthamiana*.

Fig. S2. Sequence alignment of the deduced proteins of *N. benthamiana OMT*s (*NbOMT*s) with *Vitis vinifera OMT3* (*VvOMT3*).

Fig. S3. Phylogenetic analysis of *N. benthamiana* TCPs with *A. thaliana* TCPs.

Table S1. Primer sequences for plasmid constructions, qRT-PCR, and ddPCR.

Supplementary Data
