# Supplementary material for: Phytoplasma SAP11 alters 3-isobutyl-2-methoxypyrazine biosynthesis in Nicotiana benthamiana by suppressing NbOMT1
Source: J Exp Bot. 2016 Jun 8;67(14):4415–25. doi: 10.1093/jxb/erw225 (PMC5301940; doi:10.1093/jxb/erw225)
Supplement: Supplementary Data [file supp_67_14_4415__index.html]

Phytoplasma SAP11 alters 3-isobutyl-2-methoxypyrazine biosynthesis in Nicotiana benthamiana by suppressing NbOMT1 — Phytoplasma SAP11 alters 3-isobutyl-2-methoxypyrazine biosynthesis in Nicotiana benthamiana by suppressing NbOMT1 — Supplementary Data 

# Phytoplasma SAP11 alters 3-isobutyl-2-methoxypyrazine biosynthesis in *Nicotiana benthamiana* by suppressing *NbOMT1*

## Supplementary Data

Data files

- supplementary\_table\_S1.xlsx - Supplementary Data
- supplementary\_figures\_S1\_S3.pdf - Supplementary Data
