# Supplementary material for: Phytoplasma SAP11 alters 3-isobutyl-2-methoxypyrazine biosynthesis in Nicotiana benthamiana by suppressing NbOMT1
Source: J Exp Bot. 2016 Jun 8;67(14):4415–25. doi: 10.1093/jxb/erw225 (PMC5301940; doi:10.1093/jxb/erw225)
Supplement: Supplementary Data [file supp_erw225_supplementary_figures_S1_S3.pdf]

Supplementary figure legends

Figure. S1 Cryo-SEM images of trichomes on the adaxial leaf surface in WT and *SAP11*$_{CaPM}$-transgenic *N. benthamiana*. The calculated trichome density (number of trichomes per mm^2) is presented. The scale bars denote 1 mm.

Figure. S2 Sequence alignment of the deduced proteins of *N. benthamiana OMTs* (*NbOMTs*) with *Vitis vinifera OMT3* (*VvOMT3*). Identical residues are shaded in yellow, similar residues are shaded in green, conserved residues are shaded in blue, and dimerization domains are boxed in red. The DNA fragment designed for the VIGS assay corresponding to the NbOMT1 amino acid region is underlined in red.

Figure. S3 Phylogenetic analysis of *N. benthamiana* TCPs with *A. thaliana* TCPs. The analysis was conducted using the Molecular Evolutionary Genetics Analysis Version 6.0 software package. TCP amino acid sequences were obtained from NCBI and the SOL Genomics Network (http://sgn.cornell.edu) and subjected to multiple sequence alignment using the ClustalW program. Based on the sequence alignment, a phylogenetic tree was constructed via the neighbor-joining method. In total, 69 TCPs were found in *N. benthamiana*. The numbers at branch points are bootstrap values that represent the percentage of replicate trees based on 1,000 repeats. *N. benthamiana* TCPs used in co-expression assays are indicated in bold and underlined in red.

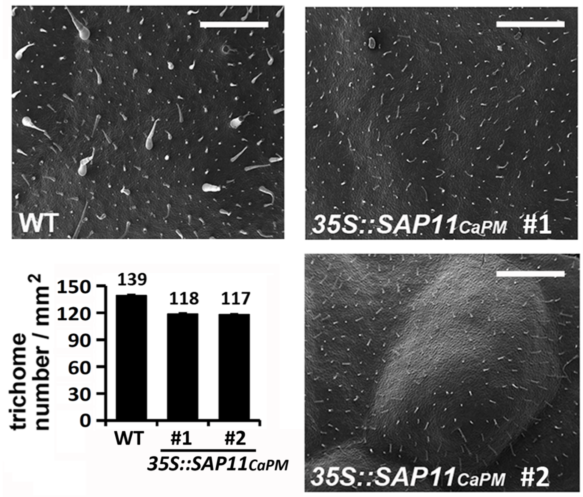
WT
35S::SAP11CaPM #1
trichome number / mm2
150
120
90
60
30
0
139
118
117
WT
#1
#2
35S::SAP11CaPM
35S::SAP11CaPM #2

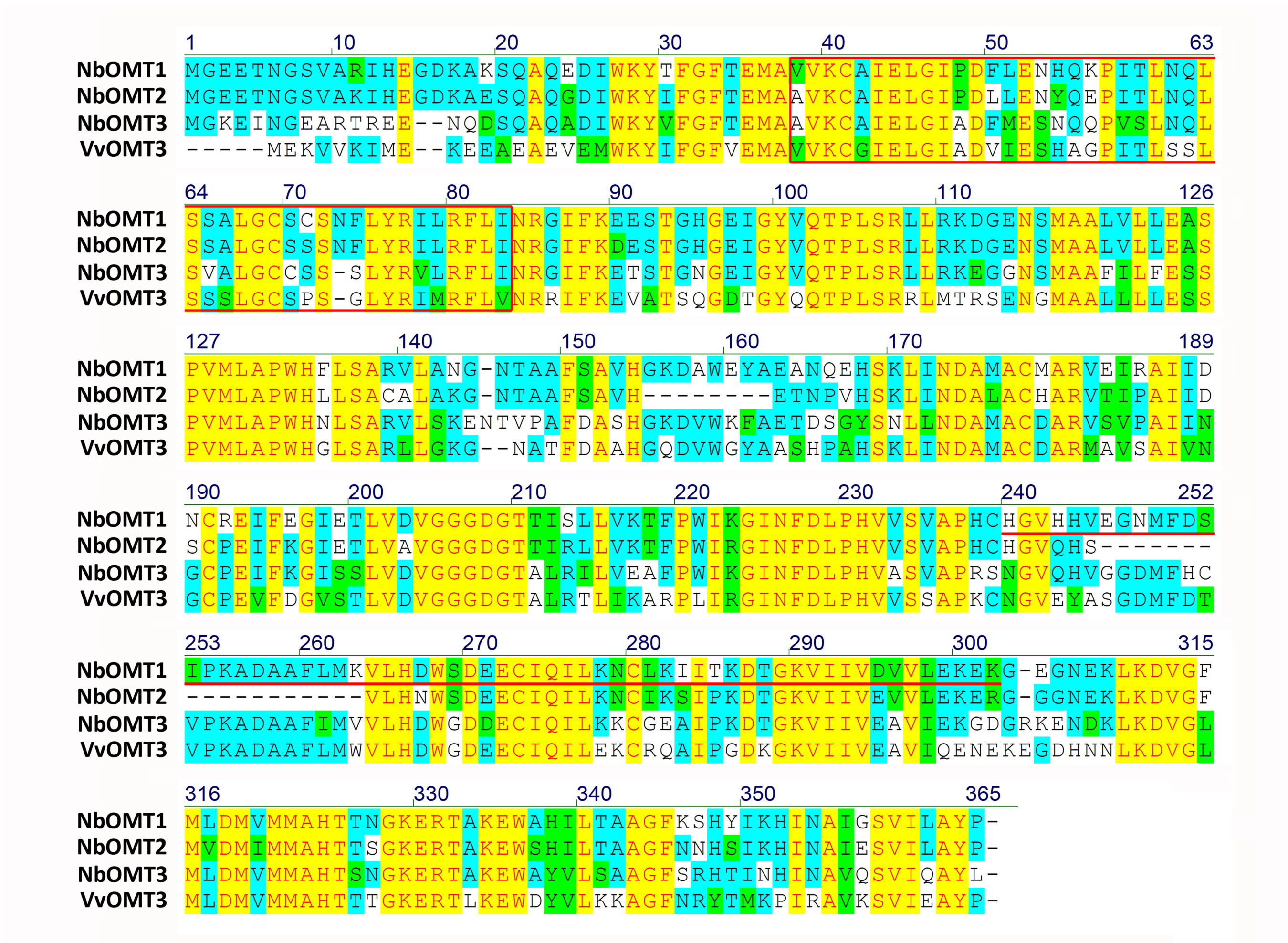

1 10 20 30 40 50 63
NbOMT1 MGEETNGSVARIHEGDKAKSQAQEDIWKYTFGFTEMAVVKCAIELGIPDFLENHQKPITLNQL
NbOMT2 MGEETNGSVAKIHEGDKAESQAQGDIWKYIFGFTEMAAVKCAIELGIPDLLENYQEPITLNQL
NbOMT3 MGKEINGEARTREE--NQDSQAQADIWKYVFGFTEMAAVKCAIELGIADFMESNQQPVSLNQL
VvOMT3 -----MEKVVKIME--KEEAEAEVEMWKYIFGFVEMAVVKCGIELGIADVIESHAGPITLSSL
64 70 80 90 100 110 126
NbOMT1 SSALGCSCSNFLYRILRFLINRGIFKEESTGHGEIGYVQTPLSRLLRKDGENSMAALVLLEAS
NbOMT2 SSALGCSSSNFLYRILRFLINRGIFKDESTGHGEIGYVQTPLSRLLRKDGENSMAALVLLEAS
NbOMT3 SVALGCCSS-SLYRVLRFLINRGIFKETSTGNGEIGYVQTPLSRLLRKEGGNSMAAFILFESS
VvOMT3 SSSLGCSPS-GLYRIMRFLVNRRIFKEVATSQGDTGYQQTPLSRRLMTRSENGMAALLLLESS
127 140 150 160 170 189
NbOMT1 PVMLAPWHFLSARVLANG-NTAAFSAVHGKDAWEYAEANQEHSKLINDAMACMARVEIRAIID
NbOMT2 PVMLAPWHLLSACALAKG-NTAAFSAVH---------ETNPVHSKLINDALACHARVTIPAIID
NbOMT3 PVMLAPWHNLSARVLSKENTVPAFDASHGKDVWKFAETDSGYSNLLNDAMACDARVSVPAIIN
VvOMT3 PVMLAPWHGLSARLLGKG--NATFDAAHGQDVWGYAASHPAHSKLINDAMACDARMAVSAIVN
190 200 210 220 230 240 252
NbOMT1 NCREIFEGIETLVDVGGGDGTTISLLVKTFPWIKGINFDLPHVVSVAPHCHGVHHVEGNMFDS
NbOMT2 SCPEIFKGIETLVAVGGGDGTTIRLLVKTFPWIRGINFDLPHVVSVAPHCHGVQHS-------
NbOMT3 GCPEIFKGISSLVDVGGGDGTALRILVEAFPWIKGINFDLPHVASVAPRSNGVQHVGGDMFHC
VvOMT3 GCPEVFDGVSTLVDVGGGDGTALRTLIKARPLIRGINFDLPHVVSSAPKCNGVEYASGDMFDT
253 260 270 280 290 300 315
NbOMT1 IPKADAAFLMKVLHDWSDEECIQILKNCLKIITKDTGKVIIVDVVLEKEKG-EGNEKLKDVGF
NbOMT2 -----------VLHNWSDEECIQILKNCIKSIPKDTGKVIIVEVVLEKERG-GGNEKLKDVGF
NbOMT3 VPKADAAFIMVVLHDWGDDECIQILKKCGEAIPKDTGKVIIVEAVIEKGDGRKENDKLKDVGL
VvOMT3 VPKADAAFLMWVLHDWGDEECIQILEKCRQAIPGDKGKVIIVEAVIQENEKEGDHNNLKDVGL
316 330 340 350 365
NbOMT1 MLDMVMMAHTTNGKERTAKEWAHILTAAGFKSHYIKHINAIGSVILAYP-
NbOMT2 MVDMIMMAHTTSGKERTAKEWSHILTAAGFNNHSIKHINAIESVILAYP-
NbOMT3 MLDMVMMAHTSNGKERTAKEWAYVLSAAGFSRHTINHINAVQSVIQAYL-
VvOMT3 MLDMVMMAHTTTGKERTLKEWDYVLKKAGFNRYTMKPIRAVKSVIEAYP-

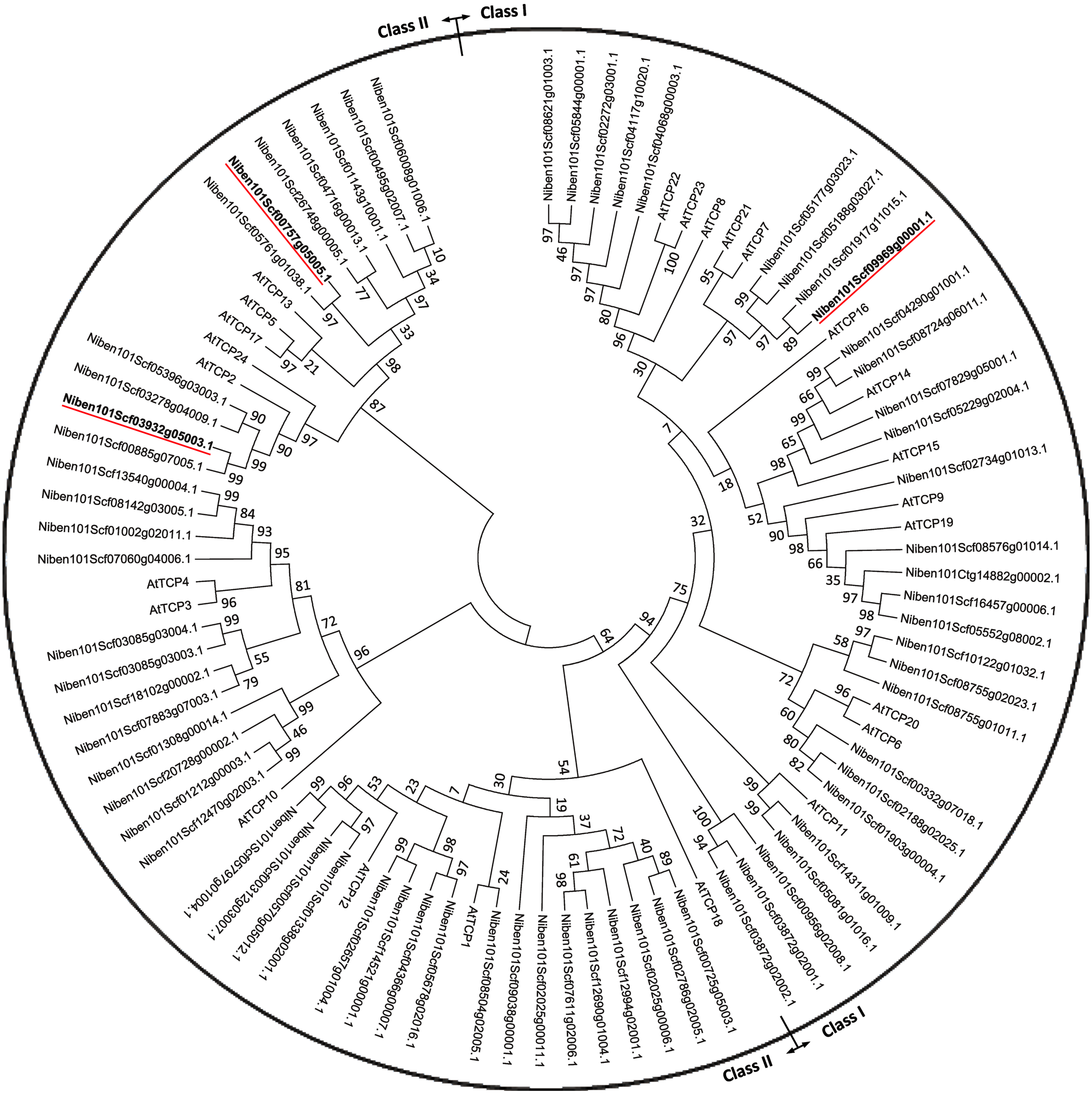
Class II
Class I
Class I
Class II
